# Spatial clustering of fatal, and non-fatal, suicide in new South Wales, Australia: implications for evidence-based prevention

**DOI:** 10.1186/s12888-017-1504-y

**Published:** 2017-10-06

**Authors:** Michelle Torok, Paul Konings, Philip J. Batterham, Helen Christensen

**Affiliations:** 1 Black Dog Institute, University of NSW, Sydney, Australia; 2 National Centre for Geographic Resources & Analysis in Primary Health Care, Australian National University, Canberra, Australia; 3 Centre for Mental Health Research, Australian National University, Canberra, Australia

**Keywords:** Suicide, Clusters, Mapping, Prevention, Scan statistics, GIS, Spatial, Epidemiology

## Abstract

**Background:**

Rates of suicide appear to be increasing, indicating a critical need for more effective prevention initiatives. To increase the efficacy of future prevention initiatives, we examined the spatial distribution of suicide deaths and suicide attempts in New South Wales (NSW), Australia, to identify where high incidence ‘suicide clusters’ were occurring. Such clusters represent candidate regions where intervention is critically needed, and likely to have the greatest impact, thus providing an evidence-base for the targeted prioritisation of resources.

**Methods:**

Analysis is based on official suicide mortality statistics for NSW, provided by the Australian Bureau of Statistics, and hospital separations for non-fatal intentional self-harm, provided through the NSW Health Admitted Patient Data Collection at a Statistical Area 2 (SA2) geography. Geographical Information System (GIS) techniques were applied to detect suicide clusters occurring between 2005 and 2013 (aggregated), for persons aged over 5 years. The final dataset contained 5466 mortality and 86,017 non-fatal intentional self-harm cases.

**Results:**

In total, 25 Local Government Areas were identified as primary or secondary likely candidate regions for intervention. Together, these regions contained approximately 200 SA2 level suicide clusters, which represented 46% (*n* = 39,869) of hospital separations and 43% (*n* = 2330) of suicide deaths between 2005 and 2013. These clusters primarily converged on the Eastern coastal fringe of NSW.

**Conclusions:**

Crude rates of suicide deaths and intentional self-harm differed at the Local Government Areas (LGA) level in NSW. There was a tendency for primary suicide clusters to occur within metropolitan and coastal regions, rather than rural areas. The findings demonstrate the importance of taking geographical variation of suicidal behaviour into account, prior to development and implementation of prevention initiatives, so that such initiatives can target key problem areas where they are likely to have maximal impact.

## Background

Suicide remains a significant public health burden, being one of the top five leading causes of years of life lost prematurely among those aged 15 to 64 years for more half a decade in Australia [1]. While suicide mortality prevalence had been temporally stable for over two decades ($\overline{x}$: 2500 per annum) [2], Australia has recently experienced a considerable increase in the number of recorded deaths, which numbered over 3000 in 2015 [1]. This increasing national trend in suicide rates is also reflected in broader global patterns [3], highlighting a critical need for more effective suicide prevention practices.

In seeking to effectively prevent suicide, a key consideration should be that of identifying where, geographically, suicide incidence is highest, and where statistically significant clusters of suicides exist. Suicide clusters are defined as excessive numbers of suicides occurring in close temporal and/or spatial proximity [2]. By identifying these geographical phenomena, decision-makers have increased translational capacity to target resources where they are likely to have the greatest impact on suicide rates, and to be able to make such decisions *prior* to the development and implementation of preventative initiatives. However, while spatial epidemiology utilising GIS technology is established in the broader field of ‘injury’ prevention research (i.e., violence, accidents, motor vehicle) [4], currently little has been done, ecologically, to map geographical variability and patterning of suicide among the general population at global, national, or regionally more discrete levels [5]. Given that it holds considerable value as a methodology for increasing our ability to detect high-risk areas, monitor the disease burden, and identify causal mechanisms, there is significant scope for uptake of GIS to advance and innovate evidence-based suicide prevention efforts.

In Australia, there have only been six studies published to date which utilise GIS to understand the distribution of suicide in the general population; four at a national level [6–9], and two at a State level [10, 11]. While, collectively, the global literature is more robust, closer inspection reveals similarly small numbers of published papers per country. As an example, four GIS studies of suicide clusters in the general population have been published in Asia [12–15], six in England [16–21], and three in America [22–24]. The focus of such research to date has primarily been on mapping purely spatial distribution of suicides, or the spatial relationship between suicide deaths and area deprivation. The paucity of research examining associations between geocoded suicide data and factors such as temporality, social context, or individual level risk, is likely a reflection of difficulties acquiring the unit level data needed for multivariate analyses. However, the current findings generally support a higher suicide risk in rural areas as compared to urbanised areas [6, 8–10, 12, 13], highlighting the capacity for GIS to identify ecological risk specificity for suicide. Such information which has translational implications for the optimisation of interventions, and reprioritisation of resources to areas presenting with significant disease clustering.

While spatial epidemiology possesses considerable value for ensuring that suicide prevention initiatives are implemented in areas of high incidence, there is a high degree of spatial specificity which makes it difficult to generalise findings between countries. Moreover, the use of different units of area measurement within studies from the same country can lead to different outcomes, even where the same input data is used [25]. As such, there is an ongoing need for countries to undertake spatial research, with a focus on standardisation of small area analysis, to make certain that areas of high disease burden are being validly identified. Given that Australian studies have either relied on national data aggregated at broad geographies, which fail to delineate discrete, small area suicide clusters, or focused only on Queensland [9, 10], there is a need to understand where suicide clusters exist, or are forming, in other Australian States so that prevention efforts can be prioritised where ‘need’ is greatest. As no studies have yet examined the spatial distribution of suicide incidents in New South Wales (NSW), this study advances the case for evidence-based suicide prevention in the State that accounts for the single largest proportion of the total national suicide burden (26.9%) [1]. The current study also contributes more broadly to the spatial epidemiological literature by being the first study to identify suicide clusters using a combined measure of suicide mortality *and* non-fatal intentional self-harm prevalence. Given that there are 10–20 suicide attempts per suicide death [26], and that suicide attempt is the strongest risk for suicide mortality [27], the inclusion of non-fatal incident data has important implications for the detection of *emerging* suicide clusters, and for timely intervention. By using the most spatially refined data to date (SA2), this study provides the most accurate representation of spatial patterns of suicide clustering in an Australian context. Specific aims of the current study were to:

1. Examine the spatial distribution of suicide deaths, and non-fatal intentional self-harm, in NSW; and
2. Identify regions at a SA2 geography, within pre-defined LGAs, which have a high relative risk of suicide mortality and non-fatal attempts as the *most likely regions* for suicide prevention intervention.

## Methods

### Design

The research design comprised a population-based retrospective study of completed suicides and hospital separations for non-fatal intentional self-harm which occurred in NSW from 2005 to 2013 (most recent data available at the time). Data was aggregated across this period to ensure sufficient numbers of suicide deaths within regions were available to enable a robust analysis; consequently, temporal cluster analysis was not possible. For all data, a SA2 nine-digit code for place of residence was used for spatial attribution. There were 548 SA2s in NSW at the time of data acquisition. Scan statistics were used to identify which SA2s formed statistically significant suicide clusters. These clusters were presented within LGA boundaries to minimise the risk of identifying individuals in areas with low counts.

### Data sources

#### Suicide mortality data

Mortality data was acquired from the Australian Bureau of Statistics (ABS). Data is provided to the ABS via the National Coronial Information System, which is used for the storage and dissemination of information about all recorded deaths in Australia. Statistics are then provided to the ABS on all suicide deaths registered, where the underlying cause of death was determined as intentional self-harm (suicide) (International Classification of Diseases, 10th edition, Australian Modification [ICD 10-AM] codes: X60-X84 [intentional self-harm], Y87.0 [sequelae of intentional self-harm]) [28]. Age and sex breakdowns at the SA2 level were not available due to confidentiality issues related to the relative infrequency of the data. For confidentiality reasons, where the total suicide deaths numbered less than five within any SA2, the ABS did not provide the number of deaths but did indicate that suicide deaths had, or had not, occurred. With the exclusion of supressed cells smaller than five individuals, 5466 deaths within 444 SA2s (out of 548, 81.0%) were available for cluster detection analysis.

### Non-fatal intentional self-harm

Data for hospital inpatient separations coded as non-fatal intentional self-harm were acquired from the NSW Admitted Patient Data Collection. Data are provided where the primary diagnosis of hospital separations was coded as intentional self-harm (suicide attempt) based on ICD 10-AM codes: X60-X84 and Y87.0. Hospital separation crude rates were calculated based on estimated residential populations. The initial data extraction included a total of 87,861 hospital separations, however, 1844 records were excluded as they did not have a valid SA2 attribute. The final dataset included 86,017 records (97.9%) for analysis.

### Spatial data

Incident data (suicide, self-harm) included SA2 codes which was linked to ABS boundary map data using ArcGIS v10.3.1. SA2s are general-purpose medium-sized areas, which aim to represent communities that interact together socially and economically. SA2s have a population range of 3000 to 25,000 persons, with an average population of approximately 10,000 persons. Only SA2s which reported > 5 suicide deaths or hospital admissions were included, such that the final dataset comprised a total of 517 SA2s.

### Statistical analysis

Two scan spatial statistic tools were used for comparative purposes, to assess differences in their utility for identifying clustering and hot-spots in NSW: (i) SaTScan [29] and (ii) Hot Spot Analysis (Getis-Ord Gi*) [30, 31]. These two spatial cluster methods were chosen as they were considered the best fit for addressing that study aims, and presented the data in the most consistent, clear, and consumable manner. SaTScan works by gradually scanning a cylindrical window with a circular base across space, adjusting for uneven geographical density of a background population. As it moves across space, it defines a collection of windows based on the total number of cases in the space. Each window represents a potential cluster which circles the centroids representing the defined census areas (SA2s). In each scan, windows are scanned from the smallest size to the maximum. We used the default values of 10%, 20% and 50% of the population for the scan parameters. Discrete Poisson probability modelling was used to compare the expected number of cases and actual number of cases inside, and outside, the window. The window with the maximum likelihood is noted as the most likely cluster (i.e. the cluster least likely to be due to chance) [29]. The system was configured to allow for geographic overlap between potential clusters, so that two or more clusters could include the same geographical space, providing that the centre of the less probable cluster was not within the area of the more likely cluster. This approach allowed for detection of neighbouring clusters that might share some suicide cases. Monte Carlo ranking with 999 replications was used to assess the strength of statistical evidence (*p* < 0.05) for the occurrence of clustering.

The second set of analyses used the Hot Spot Analysis (Getis-Ord Gi*) tool. The Gi* statistic identifies statistically significant clusters of values higher in magnitude than you might expect to find by chance. The Gi* statistic indicates whether features with high values or features with low values cluster in a specific area, by looking at each feature within the context of neighbouring features. If a feature’s value is high, and the values for all its neighbouring features are also high, it is a statistically significant ‘hot spot’. The local sum for a feature and its neighbours is compared proportionally to the sum of all features. When the local sum is much different than the expected local sum, and that difference is too large to be the result of random chance, a statistically significant z-score (*p* < 0.05) is the result. The equation for calculating the Gi* statistic is as follows:

$$ {G}_i^{\ast }=\frac{\sum_{j=1}^n{w}_{i,j}{x}_j-\overline{X}{\sum}_{j=1}^n{w}_{i,j}}{S\sqrt{\frac{\left[n\ {\sum}_{j=1}^n{w}_{i,j}^2-{\left({\sum}_{j=1}^n wi,j\right)}^2\right]}{n-1}}}\kern0.5em $$

where *x*_*j*_ is the attribute value for feature *j*, *w*_*i*, *j*_ is the spatial weight between feature *i* and *j*, *n* equals the total number of features, and:

$$ \overline{X}=\frac{\sum_{j=1}^n{x}_j}{n}\kern4.25em $$

$$ S=\sqrt{\frac{\sum_{j=1}^n{x}_j^2}{n}}-{\left(\overline{X}\right)}^2 $$

As no prior studies used both mortality and non-fatal intentional self-harm data, we sought to develop a single prevalence score to identify candidate regions with high incidence of either suicide mortality and/or intentional self-harm. No method yet exists for this purpose. To derive a single composite score, we combined and aggregated raw data rankings and cluster analyses results derived from SaTScan and Getis-ord Gi* scans. Within this approach, SaTScan cluster and Getis Ord Gi* hotspot results were allocated equal weighting in determining candidate regions. SaTScan analyses identify SA2s that represent clustering in a binary manner, such that SA2s are either in, or are not in, clusters, Hot-Spot analyses assign a Gi* statistic. Clusters with a Gi_Bin of +/−3 (Gi* z-score: <−2.58 or > + 2.58), reflect statistical significance with a 99% confidence level, areas with Gi_Bin of +/−2 (Gi* z-score: <−1.96 or > + 1.96) reflect a 95% confidence level, while areas with Gi_Bin of +/−1 (Gi* z-score: <−1.65 or > + 1.65) reflect a 90% confidence level. As the scoring functions of the two cluster tools are different we re-coded the cluster results such that each SA2 was attributed with a cluster score out of 5 as per: SA2’s identified by SaTScan and SA2s with Gi* 99% confidence score 5. SA2’s with Gi* 95% confidence score 4, SA2s with Gi* 90% confidence score 3. All other SA2s scored 0.

Determining a single composite score involved the following process:

1. The incident counts for suicide and self-harm for each SA2 were ranked using the percentrank.inc function in Microsoft Excel;
2. The crude death rates, calculated as the number of deaths registered during the reference period per 100,000 at 30 June 2011 [32], and crude hospital separation rates, calculated based on estimate residential populations for each year, for each SA2 were ranked using the percentrank.inc function in Microsoft Excel;
3. A combined Cluster Factor (CF) was calculated by averaging the two SaTScan cluster scores and Hot-Spot analyses Gi* statistics, using the following formula:
  - CF = ((SaTScan Mortality score + SaTScan Self-Harm score + HotSpot Mortality score + HotSpot Self-Harm score)/4) + 1.

These values, attributed to each SA2, were then combined to produce a single composite value referred to as the ‘SA2 composite score’. The final SA2 composite score was derived in the following manner: SA2 composite score = ((Mortality Count Rk* × 100) + (Morality Rate Rk × 100) + (Self-harm Persons Rk × 100) + (Self-harm Rate Rk × 100))/100) x CF, where ‘Rk’ refers to the PercentRank of the reference factor.

### Ethics approval

Approval for this study was obtained from the Human Research Ethics Committees of the University of New South Wales and the NSW Population and Health Services.

## Results

**Fig. 1:**
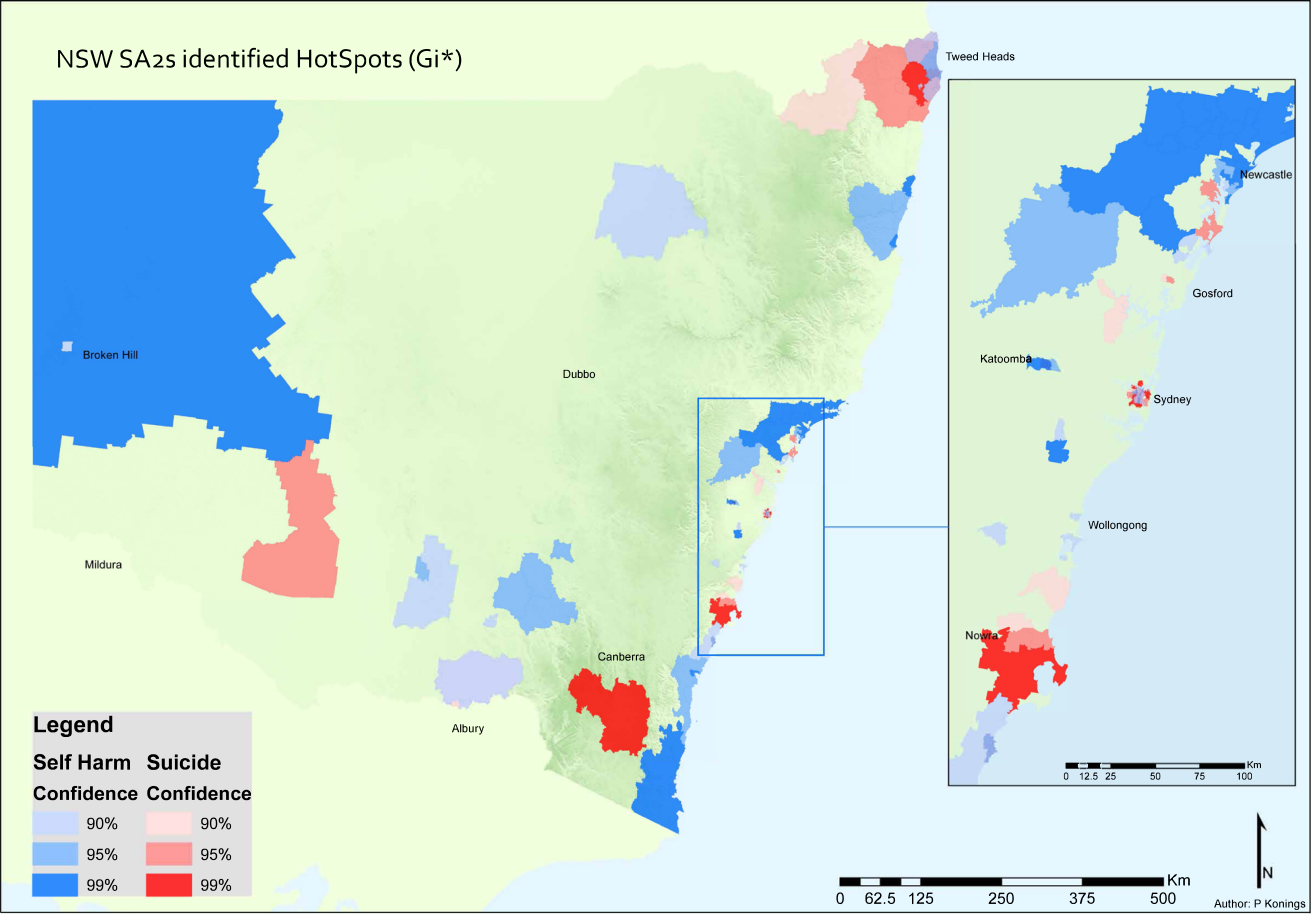
Hot Spot analyses of statistically significant suicide and self-harm spatial clusters in New South Wales

**Fig. 2:**
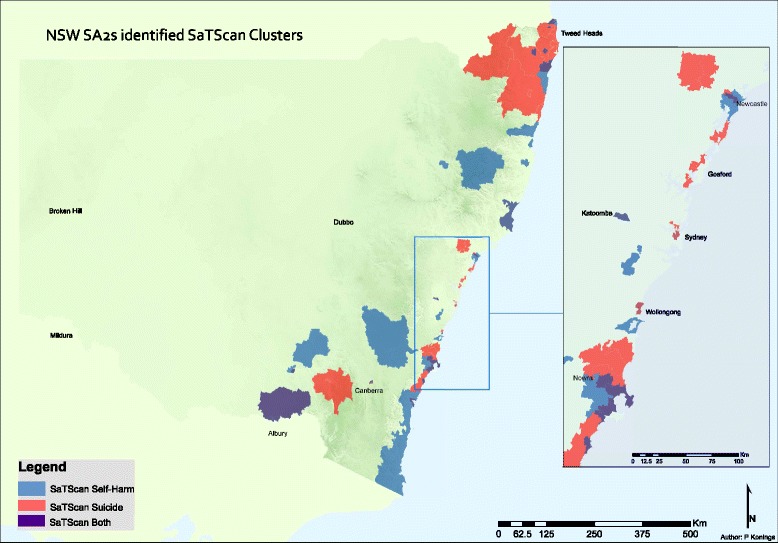
SaTScan analyses of statistically significant suicide and self-harm spatial clusters in New South Wales

Hot-Spot and SaTScan cluster analyses identified clusters of SA2s that represent regions of high incidence of suicide mortality and self-harm (i.e., in the 90th percentile), and concorded them to LGAs for reporting. Across NSW, 14.3% (*N* = 74) of SA2s identified as members of SaTScan suicide mortality clusters, 14.5% (*N* = 75) identified as suicide mortality Hot-Spots, 17.4% (*N* = 90) as members of SaTScan self-harm clusters, and, 23.8% (*N* = 123) as self-harm Hot-Spots (Figs. 1 and 2). More broadly, 40 SA2s were identified as having no self-harm cluster factor, but did have a mortality cluster factor, while 92 SA2s had no mortality cluster factor but did have a self-harm cluster factor. Comparison of the two scan statistics shows that Hot-Spot analysis identified more suicide clusters in rural or remote areas of NSW (Fig. 1), 62% of which identified as non-fatal intentional self-harm clusters. In contrast, SaTScan results indicated that clusters primarily converged on the Eastern coastal fringe of NSW (Fig. 2), which were nearly evenly split across mortality and self-harm clusters (i.e. mortality clusters 45% v. self-harm clusters 55%). Of note, Figs. 1 and 2 demonstrate minimal overlap in clusters presenting with both high suicide mortality rates *and* high intentional self-harm cluster factors.

**Fig. 3:**
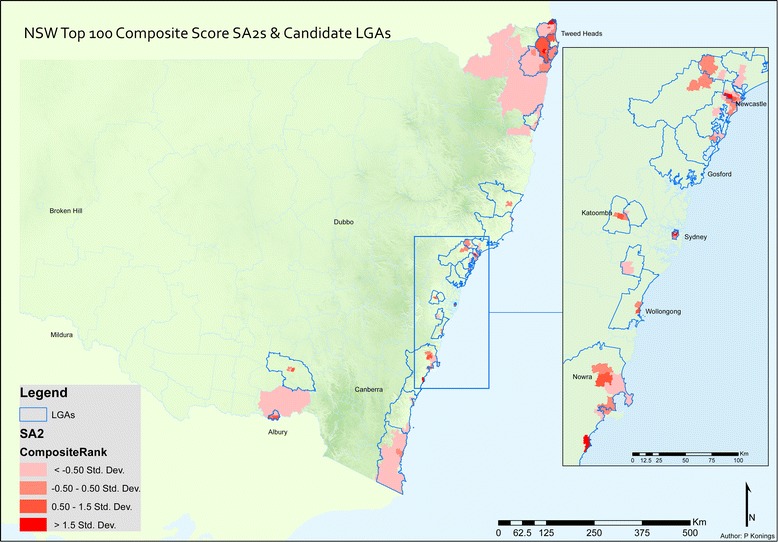
Top 100 statistically significant suicide clusters contained within 25 primary and secondary likely candidate Local Government Areas: mortality and intentional self-harm cases

Based on the composite scoring method, Fig. 3 indicates cluster areas of high suicide mortality and/or high self-harm risk in NSW. Composite scores for all SA2 clusters ranged from 0 to 22.34 ($\overline{x}$=4.0, Median = 2.3, SD = 4.2). The top 100 SA2 clusters had composite scores above 6.75, while 49 clusters scored above 10.85, representing the 90th percentile. 38.3% of all SA2s had cluster factor score above 1, meaning that they were a cluster or hot-spot candidate. These were aggregated across LGAs. The top 25 candidate LGAs (of 152, 16.4%) contained 200 SA2 hot spots, which represented 46% of hospital admissions (*N* = 39,869) and 43% of suicide deaths (*N* = 2330) between 2005 and 2013. The 25 LGA catchment areas represent the most likely candidate regions for intervention (Fig. 3).

Five spatially disparate parts of NSW were identified through composite spatial cluster analysis. As Fig. 3 shows, suicide (fatal/non-fatal) clusters aggregated in: (i) Murrumbidgee region (including Wagga Wagga and surrounds) (rural), (ii) Illawarra/Shoalhaven (coastal; Wollongong and surrounds), (iii) Sydney (metropolitan), (iv) Central Coast region (coastal; consisting of Gosford/Wyong), and (v) North Coast NSW (coastal; consisting of Byron Bay/Ballina/Lismore/Tweed Heads). There is a general increasing gradient of suicide relative risk from inland areas to the coastal areas where population density is greater, indicating a positive association between urbanicity and suicide (fatal/non-fatal) risk. No statistically significant spatial clusters were identified in remote areas of NSW.

In total, nine ‘primary clusters’ (i.e., clusters with the highest risk suicides existing close together, geographically) and 16 ‘secondary clusters’ (i.e., other high-risk clusters with significance) were identified as areas of highest risk (Table 1). Together, these 25 spatial clusters cover 5.2% of the entire study area (total geography of NSW: 809,444 km^2^), but 34.5% of the total population (based on a total population of 7.21 million) and 40.9% of total suicides (*N* = 5692 suicide in NSW for 2005–2013 aggregated) [1]. On average, secondary likely clusters had markedly lower population densities than primary clusters (75% of secondary clusters has population densities of <100 persons/km^2^ v. 22% within primary clusters), and were more likely to be categorised as rural/remote areas. Only 8% (*N* = 2) of the 25 clusters had median Socio-economic Indexes for Area (SEIFA) scores at the LGA level higher than the national average (i.e., 1000), indicating low socioeconomic status among suicide clusters. SEIFA scores were, on average, higher within the primary cluster group ($\overline{x}$: 978) than secondary cluster group ($\overline{x}$: 953), indicating greater disadvantage among the latter group.

**Table 1:** Most likely spatial clusters of suicide in NSW for data aggregated from 2005 to 2013

Local Government Area	ERP	Total geography area (km^2)^	Population density/km^2^	SEIFA score	Non-fatal self-harm (N)	Non-fatal self-harm crude rate per 100,000	Suicide mortality (N)	Mortality crude rate per 100,000	Composite score
Primary clusters									
Sydney (C)	174,185	25	8214.0	1051	2945	1691	246	141	165
Newcastle (C)	150,586	187	794.3	991	3436	2282	142	94	140
Shoalhaven (C)	93,857	4567	21.4	944	1760	1875	120	128	130
Lake Macquarie (C)	191,331	648	315.0	985	3271	1710	170	89	112
Tweed (A)	86,257	1321	69.9	949	1506	1746	102	118	97
Penrith (C)	180,521	405	442.0	989	3114	1725	148	82	89
Albury (C)	48,347	306	164.2	967	894	1849	63	130	66
Wollongong (C)	195,266	684	305.4	981	3092	1583	178	91	66
Wyong (A)	151,141	827	181.1	942	2155	1426	132	87	64
Secondary likely clusters									
Byron (A)	29,756	567	33.2	979	38	128	464	1559	54
Blacktown (C)	304,892	247	1254.0	974	218	72	3056	1002	53
Coffs Harbour (C)	69,285	1175	61.9	950	51	74	1340	1934	52
Lismore (C)	43,275	1290	33.2	946	50	116	737	1703	50
Ballina (A)	39,747	484	81.1	980	31	78	635	1598	44
Campbelltown (C)	147,717	312	509.4	943	117	79	2848	1928	44
Gosford (C)	164,192	940	172.8	1001	157	96	1862	1134	43
Wagga Wagga (C)	60,153	4826	13.1	987	56	93	1090	1812	41
Bega Valley (A)	32,274	6279	5.3	951	28	87	717	2222	38
Cessnock (C)	51,379	1966	25.4	922	56	109	729	1419	37
Eurobodalla (A)	36,137	3428	10.9	940	33	91	712	1970	37
Maitland (C)	68,192	392	172.1	986	58	85	892	1308	35
Richmond Valley (A)	22,263	3051	7.2	888	30	135	385	1729	34
Port Stephens (A)	65,537	979	66.2	970	43	66	963	1469	32
Great Lakes (A)	34,802	3376	10.2	920	26	75	518	1488	30
Greater Taree (C)	46,999	3730	12.5	906	37	79	748	1592	26

*(A)* Areas, *(C)* Cities, *ERP* Estimated Resident Population, *Km* ^*2*^ Square Kilometer, *SEIFA* Socio-Economic Indexes for Area

## Discussion

### Main findings

This study sought to identify geographical areas representing high rates of suicidal behaviour, recognising that the identification of priority regions of ‘need’ has a crucial role in the development of effective public health preventative interventions. Altogether, 25 primary and secondary likely LGA cluster candidate regions were identified, comprising mainly urbanised (i.e., higher density) coastal and metropolitan constellations. This finding is consistent with studies conducted in the United Kingdom and Denmark, which have similarly reported higher suicide rates in inner-city or coastal areas, particularly among females [18, 33]. Our findings, however, stand in stark contrast to other Australian [6, 9, 34] and international [12, 13, 17] studies which have detected stronger clustering in remote areas which have a high degree of socioeconomic deprivation and isolation, and increased difficulty accessing mental health services. The convergence of clustering in urbanised/coastal areas identified in the current study may, however, be an artefact of our data, given that this was the first study to include *non-fatal* suicide indicator data to inform cluster detection. As both the SaTScan and Hot-Spot analyses supported that clusters were more prevalently derived from self-harm incidents, rather than fatal events, this seems to be a feasible argument. It has been reported that females have a three- to four-fold risk of non-lethal suicide attempt compared to males [35], and considering this gender variation, it makes sense that spatial clusters in urbanised areas are derived from self-harm risk. Differences in the urban-rural association to suicide identified in this study, as compared to studies exclusively using mortality data, suggests that there is a degree of risk specificity which underpins fatal, versus, non-fatal suicide incidence. These risks need to be identified so they can be targeted within future prevention efforts to maximally impact suicide.

### Strengths and limitations

As far as authors are aware, this is the first study to explore spatial distribution of a combined measure of suicide mortality *and* morbidity (non-fatal self-harm), both globally and within Australia. As such, this study provides a more accurate depiction of clusters than has previously been available. The study also is significant in addressing a public health gap between suicide research and the translation of that knowledge to targeted prevention, insomuch that it makes suicide prevalence rates available to key decision makers at a geography that is typically not publicly available. Such information allows for the optimisation of regionally-specific decisions regarding intervention planning, resource distribution, and policy applications.

There were, however, some limitations to the analysis. Firstly, suicide mortality data likely underestimates the extent to which these deaths occur, as they can be misclassified (e.g., as ‘accidental’), or left as an open verdict in the absence of clear evidence, potentially leading to biased trend estimates. In contrast, non-fatal self-harm data typically fails to differentiate between suicide attempts *and* acts of deliberate self-harm lacking intent, resulting in potential overestimation. In addition, only individuals who were admitted to hospital for self-harm were included in the analyses, as this was the most robust data available, however, many suicide attempts do not result in hospital admissions. Secondly, precise spatial analysis of suicide clusters relies on data being acquired at a refined level. However, given that suicide is statistically infrequent, it is difficult to acquire data at unit level precision. It is likely that if unit level spatial data had been used, the significance of some suicide clusters may have changed, because different boundary definitions can lead to ‘clusters’ being artefactually identified, rather than signifying real, underlying disease patterns (known as the ‘modifiable areal unit problem’) [25]. Acquiring unit level data which can be aggregated in a way that most optimally reduces loss of information or bias should be a key goal of future research. Thirdly, 19% of SA2s were excluded from analyses, as these data were suppressed due to having five or fewer deaths during the study period. Had these SA2s been included, for the Getis Ord ‘hot spot’ analyses, they may have contributed positively as neighbours for high scoring regions, but it is unlikely that they would have either: a) become high scoring regions themselves, or b) made a low scoring region become a high-ranking region. For the SaTScan cluster analyses, similar adjacency contributory influence cannot be known, but as the excluded SA2s were effectively treated as zero incident regions, it is likely their influence would only be marginally different from the actual very low values, and thus not have manifestly changed the outcomes. Moreover, it was not possible to control for demographic, contextual, or socioeconomic conditions as such data could not be made available for this study due to a high potential of re-identification of data at the SA2 level. Subsequently, we could not comment on whether clusters had specific gender or age characteristics, or the influence of other social or individual level factors. It has, however, been shown that the excess area suicide risk would be reduced with adjustment by demographic characteristics [33, 34]. Moreover, obtaining better risk factor information would also allow for examination of risk specificity, and risk commonality, in suicidal behaviours, to be able to model where clusters might develop. Finally, we used temporally aggregated data which may have obscured the changes in the numerator (number of cases) and the denominator (population counts) during the study period. This limitation was unavoidable, as the strength of our aggregation was that more suicide cases could be obtained for analysis to reduce statistical error.

### Implications and future directions

Based on the findings of this study, several important implications arise. Foremost, this study provides clear rationale for where future suicide prevention efforts in NSW should be prioritised. These findings are particularly timely in the Australian context, where there has been a 20% increase in the reported number of suicides since 2013 [1], but also increasing commitment from Australian Commonwealth and State governments to fund suicide prevention efforts. There needs to be greater realisation of the potential utility of GIS mapping platforms a tool for ongoing monitoring and surveillance of the dynamic progression of suicidal clusters, so that early detection of, and intervention in, developing clusters can occur. To date, spatial studies have been limited by their lack of a temporal component, and greater efforts are needed to acquire data which measures temporal variation. Relatedly, the value of spatial analysis would be enhanced by access to more timely data. Currently, routinely collected data on suicide is markedly time-lagged, complicating efforts to precisely identify current spatial clusters. Spatial analyses can only provide an indication of what *may* be happening. This data gap should drive efforts towards establishing partnerships between coroners, police, hospitals, and other relevant data custodians, to make data more readily available, particularly at a localised level. The challenge of timely data should also drive innovation around establishing systems for collecting new sources of real-time geotemporal data, such as social media (e.g., Facebook, Twitter). Integrating this type of ‘real time’ data into GIS maps will require innovative analytical techniques which could lead to significant advancements in the prediction of suicidal behaviour, allowing intervention to occur before clusters develop.

## Conclusion

The findings from this study demonstrate the importance of taking geographical variation in suicide risk into account to better understand where suicide prevention efforts are urgently required, and likely to have the greatest impact, prior to resources being spent or used to develop or implement initiatives. Though this study has focused on the NSW, spatial epidemiology for the detection of suicide clusters has clinical and policy relevance well beyond Australia in respect to advancing evidence-based practices in suicide prevention.

## Abbreviations

ABS: Australian Bureau of Statistics
CF: Cluster Factor
GIS: Geographical Information System
ICD 10-AM: International Classification of Diseases, 10th edition, Australian Modification
LGA: Local Government Areas
NSW: New South Wales
SA2: Statistical Area 2
SEIFA: Socio-economic Indexes for Area
